# Prevalence and associated harm of engagement in self-asphyxial behaviours (‘choking game’) in young people: a systematic review

**DOI:** 10.1136/archdischild-2015-308187

**Published:** 2015-06-25

**Authors:** H Busse, T Harrop, D Gunnell, R Kipping

**Affiliations:** 1 School of Social and Community Medicine, University of Bristol, Bristol, UK; 2 Independent Public Health Doctor

**Keywords:** Injury Prevention, Adolescent Health, School Health, Public Health, Paediatric Practice

## Abstract

**Objective:**

To assess the prevalence of engagement in self-asphyxial (risk-taking) behaviour (SAB) (‘choking game’) and associated morbidity and mortality in children and young people up to age 20.

**Design:**

Systematic literature review.

**Search strategy:**

Electronic database search of MEDLINE, Embase, PsycINFO, CINAHL, PubMed, Web of Science Core Collection, BIOSIS citation index and the Cochrane register with no language or date limits applied. References of key papers were reviewed, and experts were contacted to identify additional relevant papers.

**Eligibility criteria:**

Systematic reviews, cross-sectional, cohort and case–control studies, and case reports examining SAB with regard to individuals aged 0–20 years, without explicitly stated autoerotic, suicidal or self-harm intentions were included.

**Results:**

Thirty-six relevant studies were identified, and SAB was reported in 10 countries. In North America, France and Colombia, awareness of SAB ranged from 36% to 91% across studies/settings, and the median lifetime prevalence of engagement in SAB was 7.4%. Six studies identified the potential for SAB to be associated with engagement in other risk behaviours. Ninety-nine fatal cases were reported. Of the 24 cases described in detail, most occurred when individuals engaged in SAB alone and used a ligature.

**Conclusions:**

The current evidence on SAB among young people is limited, and stems predominantly from North America and France. Awareness of SAB among young people is high, and engagement varies by setting. Further research is needed to understand the level of risk and harm associated with SAB, and to determine the appropriate public health response.

**What is already known on this topic?**

- Engagement of young people in self-asphyxial behaviour (SAB) is dangerous, and can be fatal.
- Young people engage in SAB in groups with their friends, but some continue the practice on their own.
- Despite SAB being around for decades, there is limited and little consistent evidence about the prevalence, associated risk factors and levels of morbidity and mortality associated with engagement in SAB.

**What this study adds?**

- The median lifetime prevalence rate of ever engagement in SAB in young people is 7.4% in the included cross-sectional studies from North America, France and Colombia.
- Fatal cases due to SAB have been formally reported in 10 countries around the world. Most fatal cases seem to occur when individuals engage in SAB on their own, and use ligaments to engage in the practice.
- Individuals engaging in other risk behaviours were seen to be more likely to engage in SAB, which is in line with the literature on multiple risk behaviours, which are shown to cluster and co-occur in adolescence.

## Introduction

Adolescence is a period of increased susceptibility for engaging in a range of risk behaviours such as binge drinking, unprotected sex and recreational drug use.^1^ ^2^ One less well-reported and researched form of risk behaviour in young people is engagement in self-asphyxial behaviour (SAB),^3^ also known as the ‘choking game’.^4–7^ SAB is defined as ‘self-strangulation or strangulation by another person with the hands or a noose to achieve a brief euphoric state caused by cerebral hypoxia’.^8^ A variety of methods are used to achieve the state of unconsciousness, including hyperventilation, strangulation, chest and neck compression or ligatures such as ropes or scarves.^9–13^ Various negative short-term and long-term health outcomes from engagement in SAB have been reported, including chronic headaches, confusion, amnesia, neurological damage and death.^14–17^

Engagement in SAB is not a new phenomenon. It was reported in the British Medical Journal in 1951,^18^ and similar sorts of activities are known internationally.^15^ ^19–23^ SAB is mainly referred to as the ‘choking game’ in the literature despite the existence of an extensive list of other culture-specific and language-specific terms (see online supplementary file A). The main motives for engagement in SAB are reported to be fitting in with a social group, thrill-seeking and experimentation.^24^ ^25^ These are argued to be distinctly different from self-harm, suicidal intentions and sexual asphyxia also known as autoerotic asphyxiation.^10^ ^17^ ^26^

Despite SAB being documented in the medical literature, limited epidemiological data are available on the prevalence of SAB and associated risk behaviours. Prevalence estimates are mainly from cross-sectional surveys undertaken in North America, and vary in their findings.^27–30^ The literature reports on a limited number of fatal cases due to SAB; however, advocacy groups suggest that the number of fatalities is more than 1000 worldwide.^31^ ^32^

To our knowledge, there have been no comprehensive reviews of the evidence to assess the prevalence and associated risk factors of engagement in SAB in young people. The only review we are aware of compared the clinical and psychopathological data of SAB with sexual asphyxia.^17^ It includes limited information on the frequency and associated risk behaviours of SAB.

The objective of the present review is to systematically assess the prevalence of awareness, engagement, associated morbidity and mortality in SAB in young people aged 0–20 years. We conducted the review in line with the Preferred Reporting Items for Systematic Reviews and Meta-Analyses statement (PRISMA).^33^

## Methods

### Search strategy

A systematic search was carried out in July 2014 using a predefined search protocol registered on the PROSPERO database.^34^ Neither date nor language restrictions were applied. The following eight databases were searched: MEDLINE, Embase and PsycINFO, CINAHL, PubMed, Web of Science Core Collection, BIOSIS citation index and the Cochrane Library. The search strategy was tested for effective retrieval of key papers prior to the actual search (see online supplementary file A).

**Figure 1:**
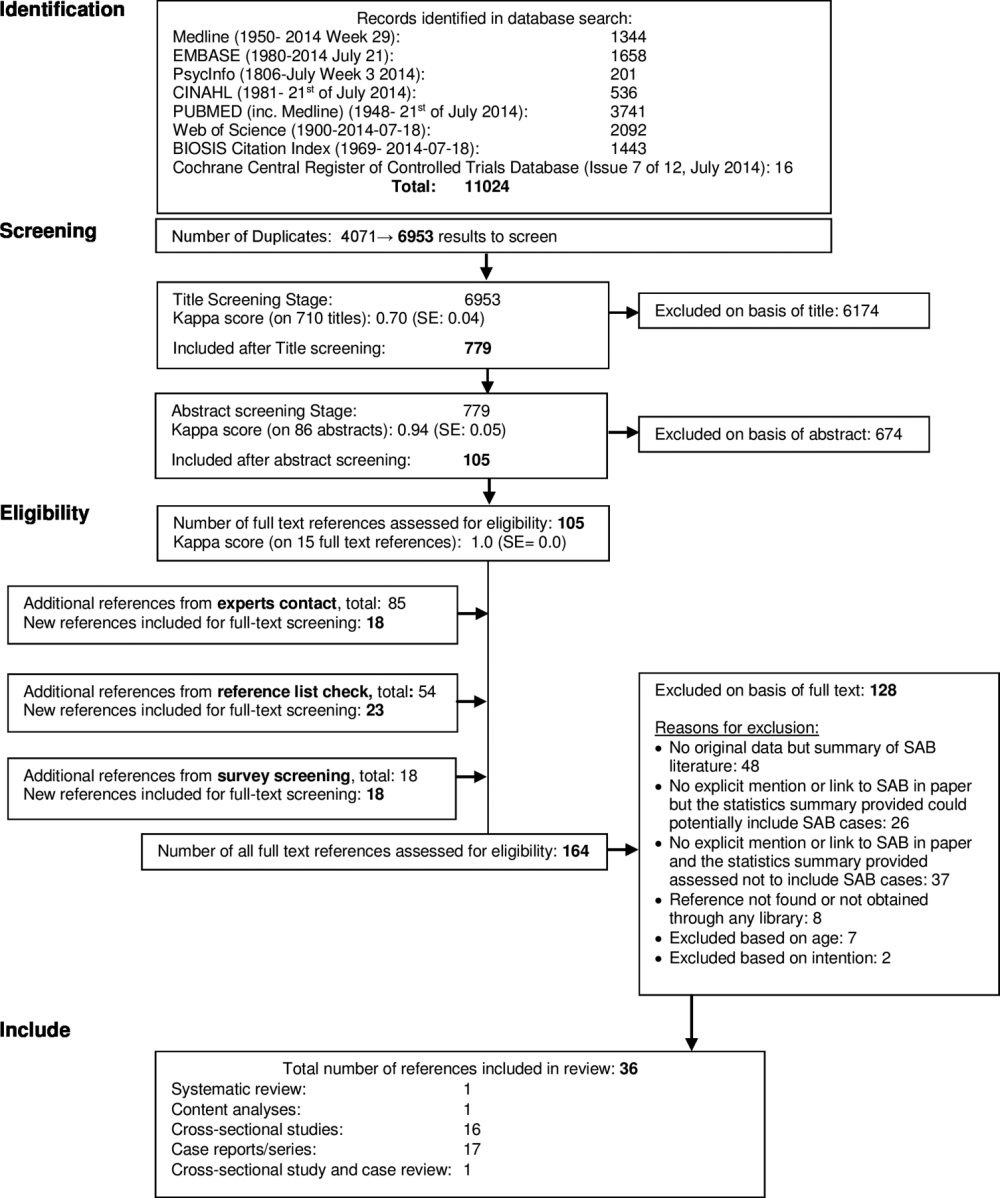
PRISMA flow chart of study search and selection process. PRISMA, Preferred Reporting Items of Systematic Reviews and Meta-Analyses; SAB, self-asphyxial behaviour.

All titles and abstracts retrieved through the searches were saved using EndNote X7 reference manager software. Duplicates were removed. Titles, abstracts and full-text references were screened for inclusion by one author with a random subset of 10% screened by a second author at each stage. Inter-rater reliability scores were calculated using Cohen's kappa, and a high level of agreement was found at all stages (figure 1). Discrepancies between reviewers were resolved by discussion.

In addition to the database search, 13 experts in the field were contacted and reference lists of 23 key SAB publications, two known SAB websites (http://www.jeudufoulard.com/ and http://www.rememberingcolin.com), and all included cross-sectional studies were hand-searched and screened to identify additional relevant studies.

### Eligibility criteria

To be considered for inclusion in the review, studies had to be either systematic reviews or provide original data on young people's engagement in SAB with the intention of undertaking an activity or a game. Any methods and settings of engagement in SAB were eligible for inclusion. Studies, where the intention to engage in SAB was described as autoerotic, or for self-harming with or without suicidal intent, were excluded.

The focus was on studies of children and young people aged 0–20 years. The age cut-off for relevant cases was 20 years, and no lower age limit was applied.^24^ ^35–37^

### Data extraction

Included studies were categorised by study design. Data extraction was carried out using predesigned data extraction forms for each study design. Data were extracted on the key study characteristics, design, methods of data collection, participant characteristics, results and conclusions drawn by authors.

### Quality assessment

Alongside the data extraction, two reviewers assessed the quality using a predetermined assessment form (available from authors). Systematic reviews were quality appraised using A MeaSurement Tool to Assess systematic Reviews (AMSTAR).^38^ To assess the quality of content analysis studies, authors created 11 appraisal questions based on the literature on content analysis and an adaptation of Crombie's critical appraisal guide.^39–41^ The quality appraisal of cross-sectional studies was based on an adaptation of the Risk of Bias Assessment Tool for Nonrandomized Studies^42^ and Crombie's critical appraisal guide.^39^ Quality appraisal of case studies and case series was based on a short array of questions informed by the Strengthening the Reporting of Observational Studies in Epidemiology statement.^43^

## Results

The search yielded 11 024 results, relating to 6953 different papers after the removal of duplicates, of which 164 references were assessed in full-text (see figure 1). Thirty-six references were included: 1 systematic review, 1 content analysis, 16 cross-sectional studies, 17 case reports/series and 1 study providing both cross-sectional and case-series data (see online supplementary file B). Almost two-thirds of studies were conducted in the USA or Canada. We found substantial heterogeneity in the studies, and therefore, decided to conduct a narrative analysis.

### Quality of studies

**Figure 2:**
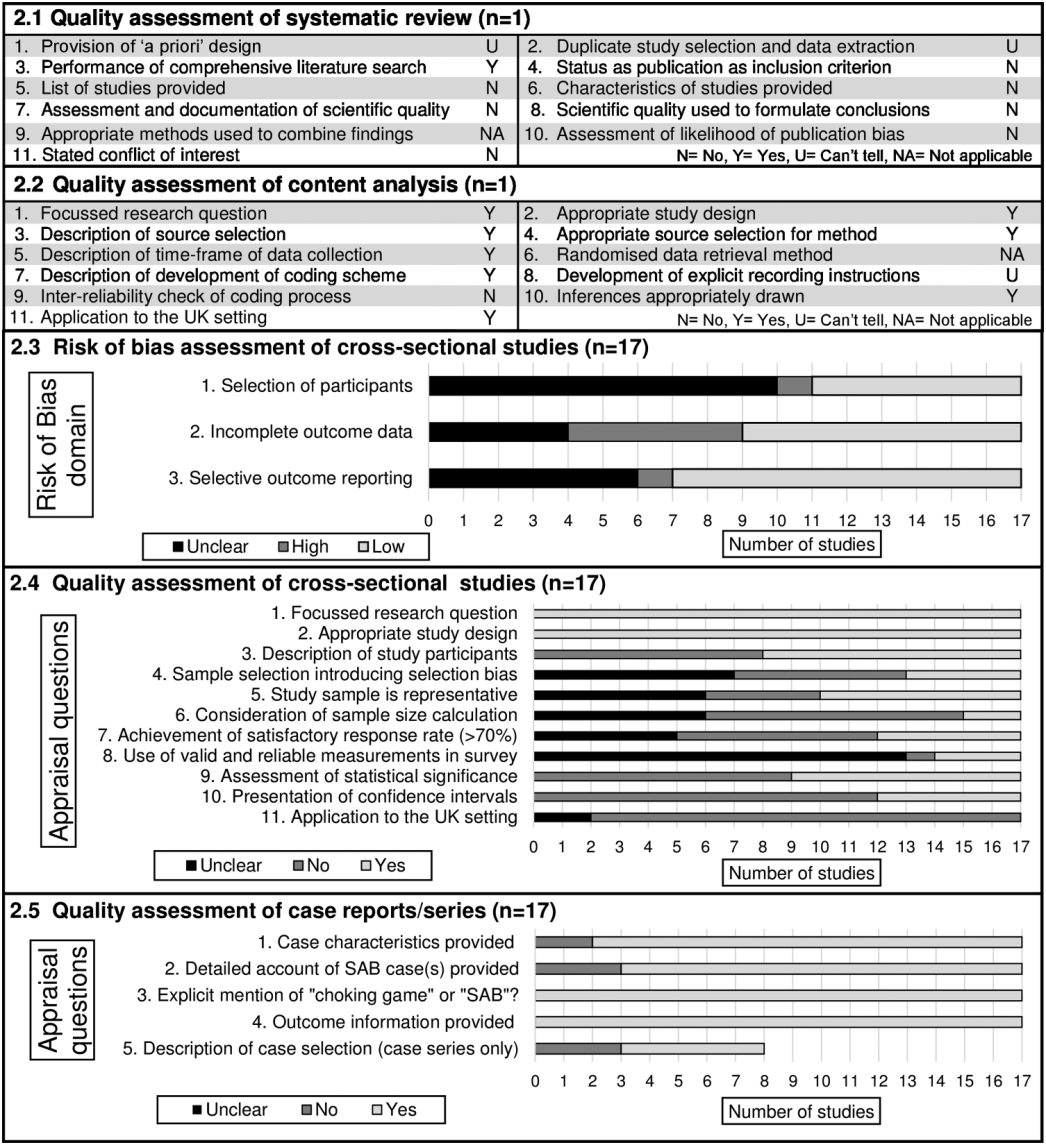
Findings from quality assessment of included studies. SAB, self-asphyxial behaviour.

A summary of quality assessment results is presented in figure 2. The systematic review was of uncertain quality as limited information was provided on its methodology; the content analysis was of high quality as methods of source selection and coding were thoroughly described. Only four cross-sectional studies were considered to be of good overall quality,^28–30^ ^36^ and many studies lacked detail on their methodology. The majority of case reports/series included sufficient details of the demographics, settings and clinical findings of the reported cases. No studies were excluded based on their quality in order to provide an overview of all the literature on SAB.

### Systematic review

The systematic review^17^ assessed and compared the clinical and psychopathological features of SAB and erotic asphyxiation in studies published between 1988 and 2011. The review included 17 references on SAB, four of which refer to original studies, which are also included in the present review. Authors suggest that SAB and erotic asphyxiation can carry addictive properties, and advise clinicians to educate themselves on the characteristics and warning signs of these behaviours.^17^

### Content analysis

One relevant content analysis was identified;^12^ it investigated videos showing recreational partial asphyxiation published in the video-sharing website *YouTube* in 2007. Sixty-five SAB videos were included with 110 participants (90% male) of estimated age between 12 and 18 years and of mixed ethnicity. Hypoxic seizures were seen in over half (55%) the videos.^12^

### Cross-sectional studies

Seventeen cross-sectional studies were included. These studies were conducted between 2007 and 2012 in four countries: US A (n=9), France (n=4), Canada (n=3) and Colombia (n=1) (table 1). Nine surveys were part of a general school health assessment, and seven aimed to obtain in-depth knowledge about SAB. The majority used self-report questionnaires, and one conducted interviews.^45^ Participants were predominantly aged 12–17, and response rates ranged from 62.2%^46^ to 95.6%.^28^

**Table 1:** Cross-sectional studies: data collection and survey details

Study details		Data collection details			Sample characteristics	
Author, year	Country (state)	Name of larger survey (if applicable)	Student response rate (%)	Number of participants in total sample (schools/classroom)	Mean age (SD), range	Gender (%Female, %Male, %Non-response)
Bernadet *et al*, 2012^46^	France		62.2	832 (7 schools)	Students aged 11–15‡	NA
Besnard and Ponroy 2004^44^	France		NA	194 (2 schools)	14.42 (0.88), 12–17	53.6, 46.4
Bonnelye, 2007^45^	France		NA	489	11.7 (3.3), 7–17	45, 55
Brausch *et al*, 2011^3^*	USA (Illinois)	Illinois Youth Survey (IYS)	65	4693 (27 schools)	16.1 (1.12), 14–19	49, 45, 6
Center for Addiction and Mental Health (CAMH), 2008^27^	Canada (Ontario)	Ontario Student Drug Use and Health Survey (OSDUHS)	68	6323 (119 schools, 385 classrooms)	Students aged 12–17‡	NA
Center for Addiction and Mental Health (CAMH), 2010^47^	Canada (Ontario)	Ontario Student Drug Use and Health Survey (OSDUHS)	65	9112 (181 schools, 573 classrooms)	Students aged 12–17‡	NA
Centers for Disease Control and Prevention (CDC), 2010^36^	USA (Oregon)	Oregon Healthy Teens (OHT) Survey	77.0 (schools), 83.7 (students)	10 642 (114 schools)	13.7 (0.5), 12–15	51.5, 48.5
Dake *et al*, 2010^29^†	USA (Ohio)		95	3598 (88 schools, 192 classrooms)	12–18 years	53, 48 (middle school); 48, 52 (high school)
Diaz Jimenez and Valencia 2014^48^	Colombia (Cali)		NA	350 (4 schools)	Students aged 12–17	57, 43
Hillard, 2012^49^*	USA (Illinois)	Illinois Youth Survey (IYS)	70	3933	14.7 (year-9 students) 17.7 (year-12 students)	50.7, 47.2, 2.1
IPSOS, 2012^50^	France		NA	1012	6–15 years	NA
Macnab *et al*, 2009^30^	Canada (Ontario) and USA (Texas)		90.7	2504 (8 schools)	13.7 (2.2), 9–18	52, 48
Maine Department of Health and Human Services and Maine Department of Education, 2012^51^	USA (Maine)	Maine Integrated Youth Health Survey (MIYHS)	71.7 (middle school), 66.7 (high school)	60 380 (325 schools)	10–18 years‡	NA
Oregon Health Authority, 2014^52^	USA (Oregon)	Oregon Healthy Teens (OHT) Survey	NA	26 731	12–18 years‡	Year 8: 50.4, 49.6; year 11: 50.6, 49.4
Ramowski *et al*, 2012^28^	USA (Oregon)	Oregon Healthy Teens (OHT) Survey	95.6	5348	12–15 years	NA
Williams County Family and Children First Council, 2007^53^	USA (Ohio)	Williams County Youth Health Assessment	97	367 (8 schools)	12–18 years	NA
Williams County Family and Children First Council, 2010^54^†	USA (Ohio)	Williams County Youth Health Assessment	95	422 (11 schools)	12–18 years	NA

*Brausch *et al* (2011)^3^ undertakes secondary analysis of Hillard (2012)^49^ data.

†Dake e*t al* (2010)^29^ seems to have incorporated Williams Country Family and Children First Council (2010).^54^

‡Specific ages estimated by authors based on stated school years.

NA, not available.

The lifetime prevalence of engaging in SAB ranged from 6% to 16% in France, 5.3%–7.4% in Canada and 3.8%–17.1% in the USA (table 2). The only Colombian study reported a prevalence of 54%.^48^ The median lifetime prevalence rate based on all available prevalence rates is 7.4%. Current participation was below 5% in most studies.^44^ ^46^ When asking young people about their knowledge of others’ engagement in SAB, prevalence rates ranged from 18.8%^52^ to 45%.^30^ Awareness of SAB ranged from 36.2%^36^ to 72%.^48^ Individuals reported first engaging in SAB when they were around 8–15 years of age,^30^ ^44^ and mentioned that they have come into contact with SAB primarily through their friends at school.^48^ ^50^

**Table 2:** Cross-sectional studies: SAB awareness and engagement

Author, year (reference)	Number of SAB questions	SAB question/definition used in survey	Lifetime prevalence of engaging in SAB % (n)	Awareness of others’ engagement	Frequency of engagement
Bernadet *et al*, 2012^46^	4*	NA	9.9 (83)	NA	NA
Besnard and Ponroy 2004^44^	8*	NA	6.7 (13)	NA	Sometimes (30.76%), weekly (15.38%), at least once a day (7.69%), no response (47%)†
Bonnelye, 2007^45^	14*	NA	12 (58)*‡	28%	NA
Brausch *et al*, 2011^3^§	1*	See Hillard, 2012^49^	16.5 (398)	NA	NA
Center for Addiction and Mental Health (CAMH), 2008^27^	1	NA	7.4 (467*)	NA	NA
Center for Addiction and Mental Health (CAMH), 2010^47^	1	Sometimes kids do risky things to ‘get high’ or to seek thrills. Have you ever been choked by someone or tried to choke yourself on purpose (like with a belt, your hands) for a short time in order to ‘get high’ or feel dizzy?	5.3 (482*)	NA	NA
CDC, 2010^36^	1	The next question refers to the ‘choking game,’ also called knock out, space monkey, flatlining or the fainting game. This is an activity that some youth participate in to get a high by cutting off blood and oxygen to the brain with a belt, towel, rope or other item. Which of the following is true for you?	5.7 (442*)	30.4%	NA
Dake *et al*, 2010^29^¶	1	Have you ever played the choking game (pass-out game, space monkey, dream game)?	5 (74) in middle school 11 (223) in high school	NA	NA
Diaz Jimenez and Valencia 2014^48^	>10*	NA	54 (190)	NA	Once (11%), twice (16%), 3 times (26%),4 or more times (47%)†
Hillard, 2012^49^	1	Have you ever been choked by someone or tried to choke yourself on purpose (like with a belt, cord or your hands) for a short time in order to get high or feel dizzy? (called the ‘choking game’)	17.1 (672*) in year 2008 13.4 (526*) in year 2010	NA	NA
IPSOS, 2012^50^	25*	Let's talk about this game where you have to hold your breath or stop your breathing. Which class were you in when you heard about this game for the first time?**	16 (161*)	32%	One time only (10%), multiple times (6%), never (84%)
Macnab *et al*, 2009^30^	8*	NA	6.6 (164)	45%	NA
Maine Department of Health and Human Services and Maine Department of Education, 2012^51^	1	Have you ever participated in the choking game or assisted another person to do so?	5.1 in middle school 7.4 in high school††,‡‡	NA	NA
Oregon Health Authority, 2014^52^	3	This is an activity that some youth participate in to get a high by cutting of blood and oxygen to the brain using a variety of methods. Which of the following is true for you?	3.9 (551*), year 8 3.8 (478*), year 11	18.8%, year 8 24.0%, year 11	None (96.5%), 1 time (1.6%), 2 times (0.9%), 3–5 times (0.4%), more than 5 times (0.6%) (year 8) None (96.5%), one time (1.7%), 2 times (0.6%), 3–5 times (0.6%), more than 5 times (0.6%) (year 11)
Ramowski *et al*, 2012^28^	2	The next question refers to the ‘choking game,’ also called knock out, space monkey, flatlining, or the fainting game. This is an activity that some youth participate in to get a high by cutting off blood and oxygen to the brain with a belt, towel, rope or other item. Which of the following is true for you? (Please mark all that apply.)	6.1, year 8 7.6, year 11††	22%, year 8 33.6%, year 11	Never (93.9%), 1 time (1.9%), 2 times (1%), 3–5 times (0.9%), more than 5 times (1.4%) (year 8) Never (91%), 1 time (3.9%), 2 or more times (5.1%) (year 11)
Williams County Family and Children First Council, 2007^53^	1*	NA	11 (40*)	NA	NA
Williams County Family and Children First Council, 2010^54^	2*	See Dake *et al*, 2010^29^	6 (25*)	NA	NA

*Number of students estimated by author based on available information and reported results.

†Based on sample of respondents who reported to have engaged in SAB.

‡Investigated engagement in dangerous games, including SAB.

§Brausch *et al* (2011)^3^ undertakes secondary analysis of Hillard (2012)^49^ data.

¶Dake *et al* (2010)^29^ seems to have incorporated Williams Country Family and Children First Council (2010) data.^54^

**Based on translation of paper.

††Exact number of participants was not able to estimate based on missing sample-size information in study.

‡‡Question asked about ever participation in SAB or assisting another person to engage in SAB.

NA, not available; SAB, self-asphyxial behaviour.

Studies generally reported that engagement in SAB is a group activity.^30^ ^50^ However, a minority of young people also reported solitary engagement in SAB, without another person present. Studies revealed that 11%^30^ ^50^–23%^44^ of young people who engaged in SAB did so without others present, and two studies reported the prevalence of solitary engagement in the respective total sample of students to range from 0.5%^52^ to 1.5%.^44^ SAB was reported to take place in a range of settings^44^ ^45^ ^48^ ^50^ and for various reasons^44^ ^45^ ^48^ ^50^ (see online supplementary file C).

When asked, young people mentioned having observed or experienced various negative health outcomes as consequences of engagement in SAB. These included having experienced headaches and dizziness,^48^ and having seen others become unconscious.^50^ Despite reporting on negative consequences of engagement in SAB, a substantial proportion of young people (17%–40%) thought that there were no risks involved.^30^ ^45^ ^48^

One study investigated the methods of prevention.^30^ Authors report that a majority of young people (57%) mentioned that knowing that SAB can lead to death or brain damage would make them stop, that younger children would most likely listen to their parents whereas older children reported to be most influenced by near-victims or peers.^30^

### Risk factors for SAB

Six out of 17 studies^3^ ^28^ ^29^ ^36^ ^44^ ^46^ reported the potential for other risk factors to be associated with engagement in SAB, and were conducted in France (n=2) and North America (n=4) (see online supplementary file D). Three of these were conducted as part of larger school surveys,^3^ ^28^ ^36^ and three were individual studies on SAB.^29^ ^44^ ^46^ Only two of the studies^28^ ^29^ explicitly mentioned controlling their analysis for possible confounding variables whereas this was not clear in all other cases, and highlights that the following section will need to be interpreted with caution. Five studies reported associations between SAB and one of the following risk-behaviour domains: substance misuse, risky sexual behaviours, poor mental health, poor dietary behaviours or engagement in risky sports.^3^ ^28^ ^29^ ^36^ ^44^

No association was found with engagement in physical activity^28^ and having experienced accidents or hospital admissions.^44^ Previous experience of violence,^28^ ^29^ being of a more impulsive and thrill-seeking personality^44^ ^46^ and lower school achievement^28^ ^29^ ^44^ were further linked to an increased likelihood of engagement in SAB. Mixed evidence was obtained with regard to gender, age and living situation.

### Case reports/series

Eighteen relevant references were included that referred to case descriptions of young people engaged in SAB (table 3). One hundred and eighty SAB cases, 99 of which were fatal, were reported in 10 of the 11 countries.

**Table 3:** Overview of included case reviews/series

Study details	Setting and data details		Cases		
Author, year	Setting	Type of data	Total cases reviewed	Fatal SAB cases/total SAB cases	Dates
Andrew and Fallon, 2007^55^	USA	Descriptive account of SAB cases	3	3/3	NA
Ayadi *et al*, 2009^56^	Tunisia	Descriptive account of SAB case	1	1/1	NA
Baquero *et al*, 2011^57^	Argentina	Descriptive account of SAB cases	8	4/4	2009, 2010
Barberia-Marcalain *et al*, 2010^11^	Spain	Descriptive account of SAB case	1	1/1	NA
Barrett, 1996^58^	USA	Descriptive account of SAB case	1	0/1	1994
Besnard and Ponroy, 2004^44^	France	Descriptive account of SAB cases	2	0/1	NA
Byard *et al*, 2011^59^	Australia	Review of cases of asphyxia	69	0/0	1994–2010
Egge *et al*, 2010^60^	USA	Descriptive account of SAB case	1	1/1	NA
Freuchen *et al*, 2012^61^	Norway	Review of suicides among young people in Norway	41	2/2	1993–2004
Gicquel *et al*, 2004^62^	France	Descriptive account of SAB case	1	0/1	NA
Klamburg Pujol *et al*, 2011^63^	Spain	Descriptive account of SAB case	1	0/1	NA
Le and Macnab *et al*, 2001^9^	Canada	Literature review to identify SAB cases using cloth towel dispensers	5	4/5	1973, 1990, 1996, 1997
McFaull 2006^64^	Canada	Literature review to identify SAB cases	74	1/74	1990–2005
Rumball, 1963^15^	UK	Descriptive account of SAB cases	2	0/3	1954, 1956
Senanayake *et al*, 2006^21^	Colombia	Descriptive account of SAB case	1	0/1	NA
Shlamovitz *et al*, 2003^22^	Israel	Descriptive account of SAB case	1	0/1	NA
Toblin *et al*, 2008^8^	USA	Newspaper and database search to identify fatal SAB cases	82	82/82	1995–2007
Ullrich *et al*, 2008^37^	USA	Descriptive account of SAB case	1	0/1	NA

NA, not available; SAB, self-asphyxial behaviour.

Two key case reviews were conducted in the USA^8^ and Canada.^64^ Toblin *et al*^8^ undertook a retrospective newspaper analysis to estimate the national incidence of deaths resulting from SAB among young people under 20 years of age between 1995 and 2007. Authors reported 82 probable SAB cases, 87% of which were males with a mean age of 13.3 years (range 6–19 years). Among 70 cases where sufficient detail was reported, 95% of cases engaged in SAB without others present. Further, most parents (93%) were not aware of SAB until their child's death.^8^ McFaull^64^ searched the Canadian injury surveillance system in 2006 in order to identify cases of asphyxia in young people, and identified 74 cases, 72% of which were males with a median age of 12.1 years (range 4–17 years). Seven cases involved solitary strangulation in which injury occurred, and one fatal case was reported.^64^

Fifteen case reports described SAB cases in sufficient detail to highlight possible risk factors for SAB (see online supplementary file E). The mean age of these 24 cases was 12.5 years (range 9–20 years), 83.3% (n=20) were male and 58% (n=14) of cases resulted in death. All of the fatal cases involved the use of ligatures, and most occurred when the individual was alone. Settings in which SAB took place were varied and included the school and home. Some fatal cases were only determined to be caused by SAB after discussions with friends or family members^8^ ^55^ ^60^ or after reviewing media content, such as from emails or phones.^55^ ^65^

## Discussion

### Main findings of this study

Thirty-six studies, the majority of which were cross-sectional and case series, were included in the review. SAB has been reported in 10 countries. The median lifetime prevalence of engagement in SAB was 7.4%. Six studies identified the potential for SAB to be associated with engagement in other risk behaviours, which is in line with the literature on multiple risk behaviours, which are shown to cluster in adolescence and to carry similar risk and protective factors.^66^ ^67^ Whereas SAB engagement usually occurs as a group activity, some individuals engage in SAB on their own. The prevalence of SAB engagement among young people varied widely, which suggests that SAB might cluster in certain areas and environments. There is potential for SAB engagement to spread to other areas, particularly through the use of social media, which is widely adopted by young people.^68^ Differences in prevalence estimates may also reflect different study methodologies. Similarly, awareness levels differ among young people as well as among parents and physicians.^23^ ^69^

Three cross-sectional studies were excluded as the mean age of respondents was above 20 years.^35^ ^70^ ^71^ Similar to included studies, lifetime prevalence in these studies were 4%^35^ and 16.2%.^71^

### Strengths and limitations

A strength of the review is the comprehensive search strategy, which included unpublished, grey literature to minimise publication bias. However, there may be further grey literature, which was not retrieved. Our inclusion of unpublished reports inevitably means that the quality of the studies is mixed.

A variety of descriptions of SAB were used in studies, which highlights the lack of an overall definition.^4^ ^5^ ^29^ As detailed in the quality assessment, some cross-sectional studies included non-random samples, had low response rates and used a single question to assess SAB engagement. These limitations require careful data interpretation and limit the generalisability of studies to other settings and countries. Additionally, caution needs to be taken in the assessment and interpretation of risk factors for engagement. Moreover, asphyxia cases reported in newspaper articles or media searches were acknowledged by authors to have low sensitivity and specificity;^8^ ^12^ a high proportion of reported cases might be due to other causes (eg, suicide), so estimates of the number of deaths from SAB should be interpreted with caution.

Some of the studies excluded from the review described SAB-type methods and behaviours in young people without explicitly naming this as SAB;^72^ this coupled with the fact that many fatal cases were only retrospectively linked to SAB^11^ ^55^ ^60^ highlights that there is a lack of knowledge and understanding about SAB and a risk for misclassification of cases.^16^ ^21^ ^55^ ^61^ It has been suggested that: ‘… what we are seeing in terms of children dying is only the tip of the iceberg of a major problem which to a large extent is unrecognised’.^73^

Preliminary data from the Office of National Statistics (ONS) on deaths of young people aged 11–15 years over a 10-year period (2002–2011) in England and Wales revealed that 145 deaths were categorised as ‘other accidental suffocation and strangulation’ (International classification of Diseases-10 (ICD-10) code W76), and 105 deaths were categorised as ‘hanging, strangulation and suffocation with undetermined intent’ (ICD-10 code Y20)^74^ (unpublished data). Some of these might have been due to SAB.

### Recommendations and future research

As limited published epidemiological data exist of SAB, we recommend further research is undertaken, particularly in countries where cases have been reported, but where no formal research on the prevalence has yet been undertaken. A wide range of prevalence estimates was obtained, which might be due to the use of different definitions and explanations of SAB within questionnaires, different study methodologies, questionnaire designs and levels of awareness, culture and engagement in SAB. It would be valuable to investigate the roles played by these factors in future research to help find explanations for the range of estimates. Additional approaches, such as investigating potential deaths through existing databases, for example, the Child Death Overview Panels (CDOP) or the ONS data in England, and making use of qualitative studies on SAB, should be considered. Finlay and colleagues reported reviewed cases of death of young people hanging from bunk beds based on the CDOP data in England, and reported that 27 out of 62 deaths from strangulation were from bunk beds with the potential for some of these deaths to be due to the SAB.^75^ Additionally, various education and intervention programmes are available, but none of these have yet been formally evaluated.

Collaboration and increased learning about this behaviour across countries, particularly among professional groups in contact with young people, may lead to a better and more accurate understanding of SAB.^16^ ^60^ Public health responses have emerged in some countries, but not in others.^9^ ^36^ ^76^

We consider it likely that specific intervention and prevention activities will need to be tailored to different settings. For areas in which SAB has been shown to be prevalent, current efforts are seen as inadequate.^45^ As it has been suggested that knowledge and identification of symptoms and signs of engagement in SAB could have possibly enabled early identification and possible prevention of fatal cases, we believe that clinicians, paediatricians, health professionals and teachers should receive education on the symptoms and signs of SAB.^22^ ^37^ ^65^ ^77^ The need to educate health professionals has been highlighted as awareness of SAB will enable these individuals to identify symptoms and signs and to act as educators to young people and their parents.^6^ ^69^ Discussions should include identifying who else would need to be educated about SAB, such as coroners, medical examiners, CDOP members, emergency service personnel and the police. We further recommend that more research is carried out together with young people to develop appropriate education material. In line with recommendations from others,^12^ ^76^ ^78^ we further recommend removing existing videos about SAB from the internet and ensuring that preventative website rather than promotional websites appear first on internet searches.^12^

## Conclusions

SAB engagement has been reported in 10 countries with high levels of awareness in young people and various levels of actual engagement. SAB is a potentially dangerous activity, which can be fatal. Further research is needed to understand the level of risk and harm associated with SAB and to determine appropriate education and prevention approaches.

## Supplementary Material

Web supplement

Web supplement

Web supplement

Web supplement

Web supplement

## References

[1] DuRant RH, Smith JA, Kreiter SR, et al The relationship between early age of onset of initial substance use and engaging in multiple health risk behaviors among young adolescents. Arch Pediatr Adolesc Med 1999;153:286–91. 10086407 10.1001/archpedi.153.3.286

[2] Arnett JJ Reckless behavior in adolescence: a developmental perspective. Dev Rev 1992;12:339–73. 10.1016/0273-2297(92)90013-R

[3] Brausch AM, Decker KM, Hadley AG Risk of suicidal ideation in adolescents with both self-asphyxial risk-taking behavior and non-suicidal self-injury. Suicide Life Threat Behav 2011;41:424–34. 10.1111/j.1943-278X.2011.00042.x 21631572

[4] Katz KA, Toblin RL Language matters: unintentional strangulation, strangulation activity, and the “choking game”. Arch Pediatr Adolesc Med 2009;163:93–4. 10.1001/archpediatrics.2008.517 19124713

[5] Sauvageau A The choking game: a misnomer. Pediatr Emerg Care 2010;26:965 10.1097/PEC.0b013e3181fe923b 21131816

[6] Andrew TA, Macnab AJ, Russell P Update on “the choking game”. J Pediatr 2009;155:777–80. [published Online First: Epub Date] 10.1016/j.jpeds.2009.06.043 19914429

[7] Mechling BA, Ahern NR, McGuinness TM The choking game: a risky behavior for youth. J Psychosoc Nurs Ment Health Serv 2013;51:15–20. 10.3928/02793695-20131029-01 24200913

[8] Toblin RL, Paulozzi LJ, Gilchrist J, et al Unintentional strangulation deaths from the “choking game” among youths aged 6–19 years—United States, 1995–2007. J Safety Res 2008;39: 445–8. 10.1016/j.jsr.2008.06.002 18786433

[9] Le D, Macnab AJ Self strangulation by hanging from cloth towel dispensers in Canadian schools. Inj Prev 2001;7:231–3. 10.1136/ip.7.3.231 11565991 PMC1730757

[10] Litman RE 500 psychological autopsies. J Forensic Sci 1989;34:638–46. 2661720

[11] Barberia-Marcalain E, Corrons-Perramon J, Suelves JM, et al El juego de la asfixia: un juego potencialmente mortal. Anales de Pediatria 2010;73:264–7. 10.1016/j.anpedi.2010.06.010 20678975

[12] Linkletter M, Gordon K, Dooley J The choking game and YouTube: a dangerous combination. Clin Pediatr 2010;49:274–9. 10.1177/0009922809339203 19596864

[13] Joye F The choking game and its variants: a physiopathological approach to risks in terms of mortality and subsequent effects in children and adolescents. In: Cochet F, ed. APEAS International Symposuim “Choking Game” and other Fainting games. Practices, Consequences and Prevention. Paris, France: L'Harmattan, 2010:35–40.

[14] Centers for Disease Control and Prevention. Unintentional strangulation deaths from the “choking game” among youths aged 6–19 years—United States, 1995–2007. MMWR Morb Mortal Wkly Rep 2008;57:141–4. 18272955

[15] Rumball A Pulmonary oedema with neurological symptoms after the fainting lark and mess trick. BMJ 1963;2:80–3. 10.1136/bmj.2.5349.80 13975507 PMC1872200

[16] Urkin J, Merrick J The choking game or suffocation roulette in adolescence. Int J Adolesc Med Health 2006;18:207–8. 10.1515/IJAMH.2006.18.2.207 16894858

[17] Ernoul A, Orsat M, Mesu C, et al Les jeux de non-oxygenation et l'hypoxyphilie: De l'asphyxie volontaire passagere a l'addiction. Annales Medico-Psychologiques 2012;170:231–7. 10.1016/j.amp.2012.02.021

[18] Howard P, Leathart GL, Dornhorst AC, et al The mess trick and the fainting lark. BMJ 1951;2:382–4. 10.1136/bmj.2.4728.382 14858861 PMC2069749

[19] Flobecker P, Ottosson J, Johansson L, et al Accidental deaths from asphyxia. A 10-year retrospective study from Sweden. Am J Forensic Med Pathol 1993;14:74–9. 10.1097/00000433-199303000-00018 8493976

[20] Indiaminov S, Iakh’'iaev TI, Tairov ST Sluchainoe udavlenie petlei. Sud Med Ekspert 1993;36:42–3. 8378983

[21] Senanayake MP, Chandraratne KA, de Silva TU, et al The “choking game”: self-strangulation with a belt and clothes rack. Ceylon Med J 2006;51:120. 17315591 10.4038/cmj.v51i3.1257

[22] Shlamovitz GZ, Assia A, Ben-Sira L, et al “Suffocation roulette”: a case of recurrent syncope in an adolescent boy. Ann Emerg Med 2003;41:223–6. 10.1067/mem.2003.49 12548272

[23] Bernacki JM, Davies WH Prevention of the Choking Game: parent perspectives. J Inj Violence Res 2012;4:73–8. 10.5249/jivr.v4i2.119 21502782 PMC3426904

[24] Lavaud J Fainting and suffocation practices in children: immediate and long-term consequences. In: Cochet F ed. APEAS International Symposium “Choking Game” and other Fainting Games: Practices, Consequences and Prevention. Paris, France: L'Harmattan, 2009:30–4.

[25] Le Breton D Anthropology of Vertigo and fainting. In: Cochet F, ed. APEAS International Symposium “Choking Game” and other Fainting Games: Practices, Consequences and Prevention. Paris, France: L'Harmattan, 2009:70–82.

[26] Le Heuzey MF Is there such thing as an at-risk child? The viewpoint of a child psychiatrist. In: Cochet F ed. APEAS International Symposium “Choking Game” and other Fainting Games: Practices, Consequences and Prevention. Paris, France: L'Harmattan, 2009:93–5.

[27] Center for Addiction and Mental Health. Highlights from the 2007 OSDUHS Mental Health and Well-Being Report. CAMH Population Studies eBulletin Toronto, Ontario: CAMH, 2008.

[28] Ramowski SK, Nystrom RJ, Rosenberg KD, et al Health risks of Oregon eighth-grade participants in the “choking game”: results from a population-based survey. Pediatrics 2012;129:846–51. 10.1542/peds.2011-2482 22508913

[29] Dake JA, Price JH, Kolm-Valdivia N, et al Association of adolescent choking game activity with selected risk behaviors. Acad Pediatr 2010;10:410–16. 10.1016/j.acap.2010.09.006 21075323

[30] Macnab AJ, Deevska M, Gagnon F, et al Asphyxial games or “the choking game”: a potentially fatal risk behaviour. Inj Prev 2009;15:45–9. 10.1136/ip.2008.018523 19190276

[31] GASP. Games Adolescents Shouldn't Play. Secondary Games Adolescents Shouldn't Play 2008 http://www.gaspinfo.com/en/home.html

[32] Ed4Ed4All. Ed4Ed4All—Education for Educators. Secondary Ed4Ed4All—Education for Educators. http://www.ed4ed4all.com/

[33] Moher D, Liberati A, Tetzlaff J, et al Preferred reporting items for systematic reviews and meta-analyses: the PRISMA statement. Ann Intern Med 2009;151:264–9. 10.7326/0003-4819-151-4-200908180-00135 19622511

[34] Busse H, Kipping R, Gunnell D, et al Self-asphyxial risk-taking behaviours in young people—a systematic review of the international evidence base 2014 http://www.crd.york.ac.uk/PROSPERO/display_record.asp?ID=CRD42014010027

[35] IPSOS. Notoriété et pratique du jeu du foulard. Étude Ipsos Public Affairs/A.P.E.A.S (Powerpoint presentation). Paris, France: IPSOS, 2007 http://www.jeudufoulard.com/html-fr/fram_01.html

[36] Centers for Disease Control and Prevention (CDC). “Choking game” awareness and participation among 8th graders—Oregon, 2008. [Erratum appears in MMWR Morb Mortal Wkly Rep. 2010 Jan 22;59(2):49]. MMWR Morb Mortal Wkly Rep 2010;59:1–5. 20075837

[37] Ullrich NJ, Bergin AM, Goodkin HP “The choking game”: self-induced hypoxia presenting as recurrent seizurelike events. Epilepsy Behav 2008;12:486–8. 10.1016/j.yebeh.2007.12.008 18218343

[38] Shea BJ, Grimshaw JM, Wells GA, et al Development of AMSTAR: a measurement tool to assess the methodological quality of systematic reviews. BMC Med Res Methodol 2007;7:10 10.1186/1471-2288-7-10 17302989 PMC1810543

[39] Crombie IK The pocket guide to critical appraisal. London, England: BMJ Publishing Group, 1996.

[40] Lombard M, Snyder-Duch J, Bracken CC Content analysis in mass communication: assessment and reporting of intercoder reliability. Hum Commun Res 2002;28:587–604. 10.1111/j.1468-2958.2002.tb00826.x

[41] Hsieh H-F, Shannon SE Three approaches to qualitative content analysis. Qual Health Res 2005;15:1277–88. 10.1177/1049732305276687 16204405

[42] Kim SY, Park JE, Lee YJ, et al Testing a tool for assessing the risk of bias for nonrandomized studies showed moderate reliability and promising validity. J Clin Epidemiol 2013;66:408–14. 10.1016/j.jclinepi.2012.09.016 23337781

[43] Von Elm E, Altman DG, Egger M, et al The Strengthening the Reporting of Observational Studies in Epidemiology [STROBE] statement: guidelines for reporting observational studies. J Clin Epidemiol 2008;61:344–9. 10.1016/j.jclinepi.2007.11.008 18313558

[44] Besnard E, Ponroy M Le jeu du foulard. France: Université François Rabelais—Tours, 2004 http://www.jeudufoulard.com/html-fr/fram_01.html

[45] Bonnelye G Jeu dangereux en milieu scolaire et extrascolaire: le point de vue des parents, la pratique des enfants. (Powerpoint Presentation) In: Étude TNS Healthcare Sofres, ed, France, 2007 http://asssm33.free.fr/Doc/Urgences/Jeux_dangereux_scolaires.pdf

[46] Bernadet S, Purper-Ouakil D, Michel G Typologie des jeux dangereux chez des collegiens: Vers une etude des profils psychologiques. Annales Medico-Psychologiques 2012;170:654–8. 10.1016/j.amp.2012.09.002

[47] Center for Addiction and Mental Health. Highlights from the 2009 OSDUHS Mental Health and Well-being Report. CAMH Population Studies eBulletin Toronto, Canada, 2010.

[48] Diaz Jimenez E, Valencia AM Knowledge about the choking game in adolescents in the city of Cali, Colombia. Turk Pediatri Arsivi (Turkish Arc Pediatr) 2014;48:65.

[49] Hillard G Body Electric: The art of healthy choices. East Central Illinois teen survey report. 2012 http://www.sarahbush.org/site_media/cms_page_media/127/final%20IYS%202012%20report.pdf

[50] IPSOS. Connaissance et pratiques du “ jeu du foulard” et autres jeux d'apnée ou d’évanouissement chez les enfants âgés de 6 à 15 ans. Étude Ipsos Public Affairs/A.P.E.A.S (Powerpoint presentation). Paris, France, 2012 http://www.jeudufoulard.com/html-fr/fram_01.html

[51] Maine Department of Health and Human Services, Maine Department of Education. Maine Integrated Youth Health Survey (MIYHS) 2011. Summary reports, 2012.

[52] Oregon Health Authority. 2013 Oregon Healthy Teeens State Report. Portland, Oregon, US: Division of Public Health, 2014:69.

[53] Williams County Family and Children First Council. Williams County Youth Health Risk Behavioral Survey. Fall 2006. Ohio, US. http://www.co.williams.oh.us/family%20first/williams%20final%20report%202-6-07.pdf 2007.

[54] Williams County Family and Children First Council. Williams County Youth Health Risk Behavioral Survey, Fall 2009. Ohio, US. http://www.hcno.org/pdf/counties/Williams%20Youth%20Final%20Report%20with%20Cover.pdf 2010.

[55] Andrew TA, Fallon KK Asphyxial games in children and adolescents. Am J Forensic Med Pathol 2007;28:303–7. 10.1097/PAF.0b013e318148bdb2 18043016

[56] Ayadi A, Hammami Z, Ben Amar W, et al Le jeu de foulard mortel chez l'enfant: A propos d'un cas. Journal de médecine légale droit médical 2009;52:26–30.

[57] Baquero F, Mosqueira M, Fotheringham M, et al El juego de la asfixia en la adolescencia: Entre la experimentacion y el riesgo. Archivos Argentinos de Pediatría 2011;109:59–61. 21283946 10.1590/S0325-00752011000100013

[58] Barrett DW Spinal air embolism resulting from a party game. Neurology 1996;47:298–9. 10.1212/WNL.47.1.298 8710101

[59] Byard RWA, Austin AE, van den Heuvel C Characteristics of asphyxial deaths in adolescence. J Forensic Leg Med 2011;18:107–9. 10.1016/j.jflm.2011.01.011 21420646

[60] Egge MK, Berkowitz CD, Toms C, et al The choking game: a cause of unintentional strangulation. Pediatr Emerg Care 2010;26:206–8. 10.1097/PEC.0b013e3181d1e3e3 20216282

[61] Freuchen AK, Kjelsberg E, Grøholt B Suicide or accident? A psychological autopsy study of suicide in youths under the age of 16 compared to deaths labeled as accidents. Child Adolesc Psychiatry Ment Health 2012;6:30 10.1186/1753-2000-6-30 22971572 PMC3526543

[62] Gicquel JJ, Bouhamida K, Dighiero P Complications ophtalmologiques du "jeu du foulard" chez un enfant de 12 ans. J Fr Ophtalmol 2004;27:1153–5. 10.1016/S0181-5512(04)96286-9 15687927

[63] Klamburg Pujol J, Torrabadella de Reynoso P, Gómez Durán EL, et al Fibrilación ventricular en paciente previamente sano. Revista Española de Medicina Legal 2011;37:177–8. 10.1016/S0377-4732(11)70086-4

[64] McFaull SR Injuries associated with playing asphyxiation games, 1990–2005, all ages. Data from the Canadian Hospitals Injury Reporting and Prevention Program (CHIRPP) (Poster presentation). Canadian Injury Prevention and Safety Promotion Conference; Toronto, Canada, 2006:35–6.

[65] Brausch AM Asphyxial Risk-taking (the “Choking Game”). In: Walsh BW, ed. Treating self-injury: A practical guide. 2nd edn New York, NY, USA: Guilford Press, 2012:305–13.

[66] MacArthur G, Smith M, Melotti R, et al Patterns of alcohol use and multiple risk behaviour by gender during early and late adolescence: the ALSPAC cohort. J Public Health (Oxf) 2012;34 (Suppl 1):i20–30. 10.1093/pubmed/fds006 22363027 PMC3284864

[67] Biglan A, Brennan PA, Foster SL, et al Helping adolescents at risk: Prevention of multiple problem behaviors. New York, USA: The Guildford Press, 2004.

[68] Montgomery K Youth and digital media: A policy research agenda. J Adolesc Health 2000;27:61–8. 10.1016/S1054-139X(00)00130-0 10904209

[69] McClave JL, Russell PJ, Lyren A, et al The choking game: Physician perspectives. Pediatrics 2010;125:82–7. 10.1542/peds.2009-1287 20008424

[70] Keathley RS, Sandlin JR The choking game: knowledge and awareness among college students. Res Q Exerc Sport 2013;84:A19.

[71] Smith B, Kercher G, Bouffard L The choking game. Crime Victim's Institute. Criminal Justice Center. Sam Houston State University, 2012 http://dev.cjcenter.org/_files/cvi/Choking_Game_Report.pdf

[72] Wyatt JP, Wyatt PW, Squires TJ, et al Hanging deaths in children. Am J Forensic Med Pathol 1998;19:343–6. 10.1097/00000433-199812000-00009 9885928

[73] Macnab AJ Asphyxial Games or “The Choking Game”: Evaluation of a potentially fatal risk behaviour. In: Cochet F ed. APEAS International Symposium “Choking Game” and other Fainting Games: Practices, Consequences and Prevention. Paris, France: L'Harmattan, 2009:41–5.

[74] World Health Organisation. International Statistical Classification of Diseases and Related Health Problems 10th Revision (ICD-10 Version). 2014 http://apps.who.int/classifications/icd10/browse/2014/en

[75] Finlay F, Lenton S, Fraser J O-002Deaths due to hanging in young people—the ‘choking Game’. Arch Dis Child 2014;99:A21–A22. 10.1136/archdischild-2014-307384.69

[76] Fernandez J Information and Prevention Methods instituted in various countries. Strategies and avenues for effective action at national and international levels. In: Cochet F ed. APEAS International Symposium. The “Choking Game” and other Fainting Games: Practices, Consequences and Prevention. Paris, France: L'Harmattan, 2009:177–80.

[77] Merrick J, Merrick-Kenig E The choking game revisited. Int J Adolesc Med Health 2010;22:173–5. 21061917

[78] Laffaire M-L From lack of prevention to choking game incitement. What practices law must consider? In: Cochet F, ed. APEAS International Symposium “Choking Game” and other Fainting Games: Practices, Consequences and Prevention. Paris, France: L'Harmattan, 2009:60–3.

