# Supplementary material for: Prevalence and associated harm of engagement in self-asphyxial behaviours (‘choking game’) in young people: a systematic review
Source: Arch Dis Child. 2015 Jun 25;100(12):1106–14. doi: 10.1136/archdischild-2015-308187 (PMC4680200; doi:10.1136/archdischild-2015-308187)
Supplement: Web supplement [file archdischild-2015-308187-s1.pdf]

Supplementary File A. MEDLINE search strategy

1	Asphyxia/
2	asphyxi*.tw
3	Airway obstruction/
4	Airway* adj4 obstruct*.tw
5	Brain Ischemia/
6	Brain ischemia.tw
7	exp Hypoxia, Brain/
8	Choking.tw
9	hypoxi*.tw
10	suffocat*.tw
11	strangulat*.tw
12	*Anoxia/
13	Anoxi*.tw
14	throttl*.tw
15	Hypocapnia/
16	hypocapni*.tw
17	Hyperventilation/
18	hyperventilat*.tw
19	syncop*.tw
20	Breath holding/
21	(breath adj2 hold*).tw.
22	(breath adj control).tw
23	(near adj hanging).tw
24	**Or/ 1-23**
25	choking game.af
26	fainting lark.af
27	mess trick.af
28	fainting game.af
29	riding a rocket.af
30	airplaning.af
31	American dream* game.af
32	Black out game.af
33	blackout game.af
34	bum rushing.af
35	california choke.af
36	California dreaming.af
37	California headrush.af
38	California high.af
39	california knockout.af
40	chok* out.af
41	cloud nine.af
42	cloud 9.af
43	dumbass game.af
44	dying game.af
45	flatlin* game.af
46	funky chicken.af
47	Harvey wallbanger.af
48	hyperventilation game.af
49	Indian headrush.af
50	knockout game.af
51	knock out game.af
52	pass out game.af
53	passout game.af
54	sleeper hold.af
55	space cowboy.af
56	space monkey.af
57	suffocation game.af
58	suffocation roulette.af
59	high riser.af
60	trip to heaven.af
61	rocket ride.af
62	speed dreaming.af
63	wall hit.af
64	purple dragon.af
65	five second high.af
66	5 minutes of heaven.af

67	seven minutes til heaven.af
68	acupuncture game.af
69	sleeping game.af
70	tap out.af
71	tingling game.af
72	twitching game.af
73	take me down.af
74	les jeux de non-oxygenation.af
75	reve indien.af
76	reve bleu.af
77	jeu du foulard.af
78	navette spatiale.af
79	30 secondes de Bonheur.af
80	jeu de la grenouille.af
81	jeu du cosmos.af
82	jeu du poumon.af
83	jeu du coma.af
84	jeu de la serviette.af
85	jeu de la tomate.af
86	jeux dangereux.af
87	evanouissement.af
88	intent desmayo.af
89	juego de la asfixia.af
90	Tomatenspiel.af
91	Ohnmachtspiel.af
92	Wuergespiel.af
93	Ohnmaechterlis.af
94	Halstuchspiel.af
95	Bio-Kiffen.af
96	Pilotentest.af
97	**Or/ 25-96**
98	risk taking/
99	risk tak*.tw
100	risktak*.tw
101	risk* behavio?r.tw
102	recreation/
103	recreation.tw
104	accidents/
105	accident*.tw
106	Games, experimental/
107	game*.tw
108	self-injurious behavior/
109	Self-injur*.tw
110	self inflicted.tw
111	“play and playthings”/
112	**Or/ 98-111**
113	Infant/
114	Infan*.tw
115	child/
116	child, Preschool/
117	child*.tw
118	Adolescent/
119	adolesc*.tw
120	preadolesc*.tw
121	pre-adolesc*.tw
122	Young adult/
123	young adult.tw
124	teen*.tw
125	teenager*.tw
126	sibling*.tw
127	early life.tw
128	Youth/
129	young*.tw
130	youth*.tw
131	boy*.tw
132	girl*.tw
133	Juvenile*.tw
134	minors/
135	minor*.tw
136	Pe?diatri*.tw
137	pubert*.tw

138	pubescen*.tw
139	pre-pubescen*.tw
140	prepubescen*.tw
141	prepube*.tw
142	pre-pube*.tw
143	puberty.tw
144	school*.tw
145	Primary school.tw
146	Elementary school.tw
147	Secondary school.tw
148	high school.tw
149	highschool.tw
150	pre-school*.tw
151	preschool*.tw
152	schoolchild*.tw
153	school child*.tw
154	Students/
155	student*.tw
156	undergrad*.tw
157	college.tw
158	campus*.tw
159	classroom*.tw
160	**Or/114-159**
161	**24 and 112 and 160**
162	**161 or 97**
