# Supplementary material for: Prevalence and associated harm of engagement in self-asphyxial behaviours (‘choking game’) in young people: a systematic review
Source: Arch Dis Child. 2015 Jun 25;100(12):1106–14. doi: 10.1136/archdischild-2015-308187 (PMC4680200; doi:10.1136/archdischild-2015-308187)
Supplement: Web supplement [file archdischild-2015-308187-s2.pdf]

Supplementary File B. Overview of included studies.

Type of study	Country (Language of publication)	Author, Year	Type of publication
Systematic Review	France (French)	Ernoul A, et al, 2012[17]	Journal publication [published]
Content analysis	Canada (English)	Linkletter M, et al, 2009[12]	Journal publication [published]
Case series	Australia (English)	Byard RW, et al, 2011[59]	Journal publication [published]
	Canada (English)	Le D, et al, 2001[9]	Journal publication [published]
	Canada (English)	McFaull SR, 2007[64]	Conference presentation [unpublished data, abstract available online: McFaull SR. Injuries associated with playing asphyxiation games among children and youth. The Canadian Hospitals Injury Reporting and Prevention Program (CHIRPP) 1990-2005. 2007 Canadian Injury Prevention and Safety Promotion Conference, November 2007, Toronto, Ontario. Abstract # 34 http://207.35.157.99/natconf/Poster_Abstracts_2007.pdf]
	Norway (English)	Freuchen A, et al, 2014[61]	Journal publication [published]
	UK (English)	Rumball A, 1963[15]	Journal publication [published]
	US (English)	Andrew TA, et al, 2007[55]	Journal publication [published]
	US (English)	Toblin RL, et al, 2008[8]	Journal publication [published]
	Argentina (Spanish)	Baquero F et al, 2011[57]	Journal publication [published]
Case reports	Colombia (English)	Senanayake MP, et al, 2006[21]	Journal publication [published]
	France (French)	Gicquel JJ, et al, 2004[62]	Journal publication [published]
	Israel (English)	Shlamovitz GZ, et al, 2003[22]	Journal publication [published]
	Spain (Spanish)	Barberia-Marcalain E et al, 2010[11]	Journal publication [published]
	Spain (Spanish)	Klamburg Pujol J, et al, 2011[63]	Journal publication [published]
	Tunisia (French)	Ayadi A, et al , 2009[56]	Journal publication [published]
	US (English)	Barrett DW, 1999[58]	Journal publication [published]
	US (English)	Egge MK, et al, 2010[60]	Journal publication [published]
	US (English)	Ullrich NJ, et al, 2008[37]	Journal publication [published]
Cross-sectional study	Canada (English)	Centre for Addiction and Mental Health, 2008[27]	Ebulletin [published]
	Canada (English)	Centre for Addiction and Mental Health, 2010[36]	Ebulletin [published]
	Canada (English)	Macnab AJ, et al, 2008[30]	Journal publication [published]
	Colombia (Spanish)	Diaz Jimenez E, et al, 2014[48]	Journal publication [published]
	France (French)	Bernadet S, et al, 2012[46]	Journal publication [published]
	France (French)	Bonnelye G, 2007[45]	Conference presentation [available online at: http://asssm33.free.fr/Doc/Urgences/Jeux_dangereux_scolaires.pdf]
	France (French)	IPSOS, 2012[50]	Conference presentation [available online at: http://www.ipsos.fr/sites/default/files/attachments/connaissance_et_pratique_du_jeu_du_foulard.pdf]
	US (English	Dake JA, et al, 2010[29]	Journal publication [published]
	US (English)	Brausch AM, et al, 2011[3]	Journal publication [published]
	US (English)	Centers for Disease Control and Prevention, 2010[36]	Organisational report [published]
	US (English)	Hillard G et al, 2012[49]	Organisational report [published]
	US (English)	Maine Department of Health and Human Services and Maine Department of Health, 2012[51]	Organisational report [published]
	US (English)	Oregon Health Authority, 2014[52]	Organisational report [published]
	US (English)	Ramowski SK, et al, 2012[28]	Journal publication [published]
	US (English)	Williams County Family and Children First Council, 2007[53]	Organisational report [published]
	US (English)	Williams County Family and Children First Council, 2010[54]	Organisational report [published]
Cross-sectional and case report study	France (French)	Besnard E, et al, 2012[44]	Students' Dissertation [available online at: http://www.jeudufoulard.com/html-fr/fram_01.html]
