# Supplementary material for: Prevalence and associated harm of engagement in self-asphyxial behaviours (‘choking game’) in young people: a systematic review
Source: Arch Dis Child. 2015 Jun 25;100(12):1106–14. doi: 10.1136/archdischild-2015-308187 (PMC4680200; doi:10.1136/archdischild-2015-308187)
Supplement: Web supplement [file archdischild-2015-308187-s3.pdf]

Supplementary File C. Settings and motivation/intent of engagement in SAB

	Settings	Motivation/Intention mentioned by young people in Sample
Besnard E, *et al*, 2012[44]*	"As regards to the context of this practice, in general they played it at home, at friends' houses or at school."	• Entertainment (61.53%)<br>• Curiosity (7.69%)<br>• Depression (15.38%)<br>• No response (15%)
Bonnelye G, 2007[45]* †	• At school, in the playground (73%)<br>• In their neighbourhood (46%)<br>• At an outdoor facility (19%)<br>• At a friend's house (17%)<br>• At home (16%)<br>• On summer camp (14%)<br>• On holiday with cousins or friends (10%)<br>• Don't know (13%)	• "Everyone plays them/I want to do what my friends are doing" (59%)<br>• "It's fun" (46%)<br>• "To prove my strength" (13%)<br>• "To show I'm not scared" (8%)<br>• "I felt forced to do it" (7%)
Diaz Jimenez E, *et al*, 2014[48]*	• Friend's house (50%)<br>• School (27%)	• Curiosity (55%)<br>• Experimentation (2%)<br>• Peer pressure (30%)
IPSOS, 2012[50]*	• In the playground (82%)<br>• In the canteen (15%)<br>• At school (86%)<br>• At own house (10%)<br>• In an outdoor place/on summer camp (7%)<br>• At sports club (6%)<br>• At boarding school (1%)<br>• Somewhere else (10%)<br>• Could not say (1%)	• Because everyone/friends are playing it" (50%)<br>• "It's a craze" (32%)<br>• "Because it's a funny game and it gives me strange/weird feelings" (16%)<br>• "To be part of my group of friends" (14%)<br>• "To unwind" (9%)<br>• "Could not say" (3%)

Notes: *Based on translations of the foreign language studies. †Investigated engagement in dangerous games including SAB.
