# Supplementary material for: Prevalence and associated harm of engagement in self-asphyxial behaviours (‘choking game’) in young people: a systematic review
Source: Arch Dis Child. 2015 Jun 25;100(12):1106–14. doi: 10.1136/archdischild-2015-308187 (PMC4680200; doi:10.1136/archdischild-2015-308187)
Supplement: Web supplement [file archdischild-2015-308187-s4.pdf]

Supplementary File D. Engaging in SAB and risk factors

Risk factor categories	Variables	Evidence of association (and references)	Evidence of no association
Demo-graphics	Age	• Being older [29 44]*	[3 36]
	Gender	• Being male [29] (high school students only)*	[3 28 36]
	Ethnicity	• American Indian/Alaska Native (vs. all others)[36]*<br>• Being of African American or Pacific Islander ethnicity (vs. white ethnicity) [28]*	
Living situation	Home situation	• Not living with two parents [29]*	[44]
	Geographical location	• Living in rural location [36]*	[28]
Education	School achievement	• Having repeated a school year [44]*<br>• Lower grades [28 29]*	
Health risk factors	Substance Use	• Substance use unspecified [3 36]*	
	Alcohol use	• Alcohol use [44] [28]*<br>• Binge drinking [29]*	
	Tobacco use	• Tobacco use [44] [29] [28]*	
	Cannabis and drug use	• Cannabis use [44] [29] [28]*<br>• Other (illegal) drug use [44]*	
	Inhalant/solvent use	• Ever used medications without a prescription to get high [29]<br>• Ever used inhalants to get high [29] (high school only)*	
	Mental health	• General mental health [36]*<br>• Higher levels of depression [44]<br>• Higher Disinhibition and "general feeling" scores [44]<br>• Engagement in suicidal behaviours [3]*<br>• Attempted suicide in past year [29]*<br>• Contemplated suicide [28]*<br>• Felt sad/hopeless for over two weeks in past year [29] [28]*<br>• Fair/poor mental health status/ unmet mental health need [28]*	
	Diet	• Disordered eating [3]*<br>• Poor nutrition (food insecurity, low fruit/vegetable and breakfast consumption [28]*	
	Sexual health behaviours	• Having ever been forced to have sexual intercourse [29]*<br>• Higher number of sexual partners [29]* (high school only)<br>• Ever had sexual intercourse [28]*	
	Physical Activity		[28]
	Risk behaviours	• Engagement in risky sports (e.g. roller-blading, boxing, and skate-boarding) [44]*<br>• Engagement in risky motor vehicle use (e.g. speeding, riding without a helmet,…) [44]*	
	Gambling	• Ever gambled for money, gambling in past 30 days [28]*	
	Physical health (accidents/ hospital admissions)		[28]
	Other behaviours	• Engagement in other unhealthy behaviours [36]	
Exposure factors	Exposure to violence/crime	• Experience of violence by others in past year [29]*<br>• Been threatened with a weapon [28]*<br>• Gun use in past 30 days [28]*<br>• Skipped school because felt unsafe [28]*	
Personality traits		• Borderline, Impulsivity and Antisocial personality traits [44]<br>• Raised hyperactive / impulsive symptomatology [46]<br>• Strong "novelty seeking" [46]*<br>• Use of active coping strategies (seeking advice) in resolving conflict [46]	

Notes: Only cross-sectional studies that report on the variables are displayed in the table. *Statistical significant association between risk factor and SAB reported by study author(s) at p≤0.5 level.
