# Supplementary material for: Prevalence and associated harm of engagement in self-asphyxial behaviours (‘choking game’) in young people: a systematic review
Source: Arch Dis Child. 2015 Jun 25;100(12):1106–14. doi: 10.1136/archdischild-2015-308187 (PMC4680200; doi:10.1136/archdischild-2015-308187)
Supplement: Web supplement [file archdischild-2015-308187-s5.pdf]

Supplementary File E. Case studies: detailed case descriptions

Study details	Case descriptions				
**Author, Year**	**Age, Gender**	**Use of ligature**	**Setting**	**Background**	**Fatal**
Andrew TA, *et al*, 2007[50]	9, M	Yes (thin rope)	Alone, in bedroom	Female sibling described individual as playing with rope about his neck on this and on previous occasions.	Yes
	13, M	Yes (dog leash)	Alone, in basement of own home	Mother wrote email afterwards alluding to the deceased's involvement with an asphyxial game known as “Space Monkey”.	Yes
	11, M	Yes (dog leash)	Alone, in bedroom	The deceased's intent, stated to sibling, was to increase the time in which he could remain conscious while playing.	Yes
Ayadi A, *et al* , 2009[51]*	11, M	Yes (long scarf)	Alone, at home	Students in school engaged in SAB.	Yes
Barberia-Marcalain E *et al*, 2010[9]*	15, M	Yes (noose tied to bunk bed)	Alone, in bedroom	Brother revealed that he showed deceased how to lose consciousness.	Yes
Barrett DW, 1999[53]	12, F	Yes (shoelaces)	In group, at party	At party, participants attempted to cause one another to lose consciousness.	No
Besnard E, *et al*, 2012[39]*	14, F	No	In group, at park and friends' house	Introduced to SAB by older cousins.	No
Egge MK, *et al*, 2010[55]	12, F	Yes (hanging from bunk bed)	Alone, in bedroom	Cousin revealed engaging in SAB with the deceased by pulling clothing tight around the neck to prevent oxygen flow.	Yes
Freuchen A, *et al*, 2014[56]	M	Yes (noose around neck)	Alone, at home	NA	Yes
	M	Yes (noose around neck)	Alone, at home	NA	Yes
Gicquel JJ, *et al*, 2004[57]*	12, M	NA	In school	Individual revealed engagement in SAB to physician.	No
Klamburg Pujol J, *et al*, 2011[58]*	19, M	NA	In group, at party	Friends revealed engagement in SAB to physician after incidence.	No
Le D, *et al*, 2001[7]	9, M	Yes (cloth towel from towel dispenser)	In group, in school washroom	NA	Yes
	12, M		Alone, in school washroom	NA	No
	7, M		NA	NA	Yes
	7, M		NA	NA	Yes
	11, M		NA	NA	Yes
Rumball A, 1963[13]	20, M	No	In group with friends	Individual revealed engagement in SAB and wanted to improve on the usual performance by its repetition in quick succession and without interval.	No
	19, M	No	In group, lunchroom	Individual was "persuaded" by friends of similar age to engage in SAB for the first time.	No
Senanayake MP, *et al*, 2006[19]	10, M	Yes (belt)	Alone, in bedroom	NA	No
Shlamovitz GZ, *et al*, 2003[20]	12, M	No	In group with friends	Individual reported at least 4 previous episodes and added that sometimes the game is played in a pair and that the “loser” is the one who passes out first.	No
Toblin RL, *et al*, 2008[6]	13, M	Yes (belt)	Alone, in bedroom.	Young people engaged in SAB at local parties.	Yes
	13, F	Yes (belt and shoelace)	Alone, in bedroom	Individual had confided in a cousin that she recently had played the “choking game” in the locker room at school and that a group of girls at her school had been suspended for playing this.	Yes
Ullrich NJ, *et al*, 2008[34]	14, M	No	NA	EEG-bedside monitoring showed how person place his hands on his neck to restrict the oxygen flow to the brain.	No

Note: *Based on translation of foreign language studies. NA: not available
